# Saprochaete Capitata Peritonitis in a Non-neutropenic Patient Without Underlying Malignancies: A Case Report

**DOI:** 10.7759/cureus.45568

**Published:** 2023-09-19

**Authors:** Fatima Zahra Rahali, Fatima Bounani, Fatima Babokh, Awatif El Hakkouni

**Affiliations:** 1 Department of Clinical Laboratory, Faculty of Medicine and Pharmacy, Mohammed VI University Hospital, Marrakesh, MAR; 2 Department of Biology/Department of Parasitology and Mycology, Faculty of Medicine and Pharmacy, Mohammed VI University Hospital, Cadi Ayyad University, Marrakesh, MAR

**Keywords:** antifungal therapy, non-neutropenic patient, peritonitis, magnusiomyces capitatus, saprochaete capitata

## Abstract

*Saprochaete capitata* (*S. capitata*) is an opportunistic arthroconidial yeast-like fungus causing invasive infections in immunocompromised patients, mainly those with hematological malignancies and severe neutropenia. However, infections due to *S. capitata* are extremely rare in immunocompetent and non-neutropenic patients. *Saprochaete* spp. are microscopically characterized by arthroconidia with hyaline-septated hyphae. *S. capitata* is known to be intrinsically resistant to echinocandins and highly resistant to fluconazole. It is suggested to use amphotericin B or voriconazole (in monotherapy or in combination) as the gold standard treatment for *S. capitata*systemic infections. We report a rare case of *S. capitata* peritonitis with fatal outcome in a non-neutropenic patient without underlying malignancies. This case report highlights the value of direct microscopic examination and stained smears in a prompt preliminary diagnosis of *S. capitata* invasive fungal infections. We also aim to emphasize the importance of early initiation of appropriate antifungal treatment in patients with *S. capitata* systemic infections, thus improving their therapeutic outcome.

## Introduction

*Saprochaete capitata* (*S. capitata*) is an arthroconidial yeast-like filamentous fungus [1,2]. *S. capitata* is also known as *Geotrichum capitatum*, *Magnusiomyces capitatus*, *Trichosporon capitatum*, *Blastoschizomyces capitatus,* and *Dipodascus capitatus* [1-3]. It is an Ascomycetous yeast classified in the family Dipodascaceae and belonging to the order Saccharomycetales [2,4].

*S. capitata* is an opportunistic pathogen causing invasive fungal infections (IFI) in immunocompromised patients, mainly those with hematological malignancies and severe neutropenia, with a high mortality rate (60%) [5]. However, *S. capitata* infections rarely occur in immunocompetent and non-neutropenic patients [4]. *S. capitata* is characterized by its intrinsic resistance to echinocandins with high resistance to fluconazole [1,4].

We report a rare case of *S. capitata* peritonitis with a fatal outcome in a patient without neutropenia or associated malignancy.

## Case presentation

An immunocompetent 60-year-old man with a history of gastric ulcer and chronic tobacco use was admitted to our hospital with acute abdominal pain evolving for 12 hours. Physical examination showed tachycardia (107 beats per minute), hypotension (80/50 mmHg), and fever at 39°C. Palpation revealed a distended abdomen with generalized tenderness and rigidity in all quadrants.

Blood investigations showed leukocytosis: 22.330/mm³; neutrophilia: 19.270/mm³; and raised C-reactive-protein level: 141 mg/L. The plain abdominal X-ray revealed pneumoperitoneum. The history of gastric ulcer associated with pneumoperitoneum suggested a peritonitis due to a perforated gastric ulcer. Empiric intravenous antibiotic therapy combining ceftriaxone, gentamicin, and metronidazole was initiated.

The patient underwent laparotomy, and the peritoneal fluid was sampled intraoperatively. A gastric perforation was identified and closed after decontamination, and a peritoneal lavage was performed. Postoperatively, the intravenous antibiotic therapy was continued.

**Figure 1:**
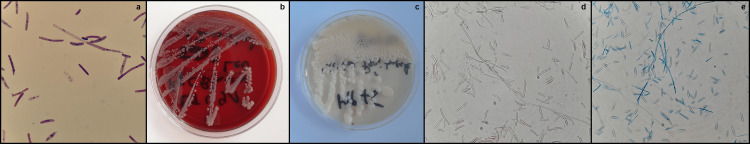
(a) Gram-stained smear from peritoneal fluid showing rectangular arthroconidia. (b and c): Culture of Saprochaete capitata on blood agar (b) and on Sabouraud-chloramphenicol agar (c) after 48 hours of incubation, showing whitish, dry, and wrinkled yeast colonies. (d and e): Wet mount preparation (d) and lactophenol blue stain (e) of culture showing rectangular arthroconidia and septated hyaline hyphae branched at acute angles.

Gram-stained smear of the peritoneal fluid showed multiple rectangular arthroconidia (Figure 1a). Culture on blood agar and Sabouraud-chloramphenicol agar at 37°C yielded whitish, dry, and wrinkled yeast colonies after 48 h (Figure 1b, 1c). The isolated yeast grew also on Sabouraud dextrose agar with cycloheximide. The urease activity of the isolate was negative. Microscopically, wet mount preparation and lactophenol blue stain of culture revealed rectangular arthroconidia and septated hyaline hyphae branched at acute angles (Figure 1d, 1e).

Three days after admission, antifungal therapy with an intravenous infusion of fluconazole was started (400 mg/day). There was no bacterial coinfection in our patient.

The isolated yeast was identified as *S. capitata* using Vitek 2 compact YST ID cards (Biomérieux) with a confidence value of 93%. Matrix-assisted laser desorption ionization-time of flight (MALDI-TOF) mass spectrometry (Microflex™; Bruker Daltonic, Bremen, Germany) identified the isolate as *Magnusiomyces capitatus* with an identification score of 2. Neither sequencing of the internal transcribed spacer (ITS) region of DNA nor antifungal susceptibility testing were performed.

On day three post-admission, the patient’s course was complicated, and he died of multiorgan failure just before the identification of isolated yeast.

## Discussion

According to the latest taxonomy revisions, *S. capitata* is an arthroconidial yeast-like fungus, related to Ascomycetes, belonging to Dipodascaceae family, Saccharomycetes order [1]. *S. capitata* has multiple former names: *Geotrichum capitatum*, *Trichosporon capitatum*, *Magnusiomyces capitatus*, *Blastoschizomyces capitatus*, and *Dipodascus capitatus* [1-3].

*S. capitata* is present in soil, sand, and wood pulp [1,5]. *S. capitata* has also been isolated from milk [1]. Gurgui et al. have reported an outbreak of *S. capitata* associated with contaminated milk in the hematology department in Spain [6]. *S. capitata* may be found in colonization in the skin, digestive, and respiratory tracts of healthy people [1,3]. *S. capitata* IFI are very rare in immunocompetent patients [7]. In immunocompromised patients, *S. capitata* is an emerging yeast responsible for life-threatening IFI, particularly in neutropenic hematological patients [3,5]. Acute myeloid leukemia (AML) patients are by far the most affected by this opportunistic pathogen [5].

The risk factors of *S. capitata* IFI are severe and profound neutropenia, the presence of a central venous catheter, the use of antibiotics with a large broad spectrum, and chemotherapy altering the gastrointestinal mucosal barrier [1,8]. Furthermore, several breakthrough infections caused by *S. capitata* have been reported in patients treated empirically with echinocandins [4,5,8,9].

In immunocompromised patients, *S. capitata* is mostly responsible for fungemia [1]. It can also affect the lungs, liver, gut, kidney, bone, and bone marrow [1,4]. *S. capitata* peritonitis are very rare [1]. In a literature review of *S. capitata* IFI, this yeast was isolated from blood culture in 75% of the cases, and from other sterile sites in the remaining 25% (cerebrospinal fluid, peritoneal fluid, urine, and tissue biopsies) [5]. Moreover, it was revealed that 87% of *S. capitata* infections were characterized by underlying hematological malignancies, mainly AML (52%) [5]. In addition, 82% of patients were neutropenic, while 13% didn’t have neutropenia [5]. In our case, the patient wasn’t neutropenic and didn’t have any underlying malignancies.

D’Assumpcao et al. have reported a case of *S. capitata* peritonitis without underlying malignancies in an adult man with necrotizing pancreatitis who survived after appropriate antifungal therapy [7]. To our knowledge, this is the first reported case of *S. capitata* peritonitis with a fatal outcome in a patient without neutropenia or associated malignancy. Bariş et al. reported a case of peritonitis due to *S. capitat*a in a child with acute lymphocytic leukemia during caspofungin therapy; the patient’s condition was improved after antifungal treatment adjustment [10].

The diagnosis of *Saprochaete *spp. infections is proven by a culture of blood or another sterile affected site [1,3]. Culture on Sabouraud-chloramphenicol agar at 37°C yielded whitish, dry, and wrinkled colonies after 24-48 h of incubation [1]. However, cultures may take a long time of up to five days, leading to diagnostic delays [1]. Generally, features allowing for differentiating *Saprochaete *spp. from *Trichoporon *spp. are urease negativity of *Saprochaete *spp. and its ability to grow on Sabouraud dextrose agar with cycloheximide [3,10]. Microscopically, *Saprochaete *spp. morphology is characterized by true fragmented hyphae, pseudo-hyphae, arthroconidia, and annelloconidia [1,3]. It is important to carefully observe the gram smears looking for arthroconidial forms, which are an essential morphological characteristic allowing early preliminary diagnosis of arthrosporic yeast infections. Furthermore, it is important to note that arthroconidia are not specific to *Saprochaete *species [4,9].

*S. capitata* identification can be made by different commercial systems such as Vitek 2 (YST ID card, Biomérieux) and Api ID 32 C (Biomérieux). However, these systems do not cover *S. clavata* species [1]. MALDI-TOF mass spectrometry is an excellent and reliable identification technique distinguishing between *S. capitata* and *S. clavata* [1,9]. Further, polymerase chain reaction assays and the sequencing of ITS are useful diagnostic tools for the discrimination of *Saprochaete *species [1].

There are no therapeutic guidelines for the treatment of *Saprochaetes *IFI [1,4,5]. *Saprochaetes *are intrinsically resistant to echinocandins and highly resistant to fluconazole [1]. It is suggested to use amphotericin B or voriconazole (in monotherapy or in combination) as the gold standard treatment in* S. capitata* IFI [1,5]. Moreover, the treatment of infections due to this organism also includes source control (drainage of retroperitoneal fluid collection and rapid removal of central venous catheters) [1,3].

## Conclusions

To our knowledge, this is the first reported case of *S. capitata* peritonitis with a fatal outcome in a non-neutropenic patient without underlying malignancies. *S. capitata* is intrinsically resistant to echinocandins and highly resistant to fluconazole. Arthroconidial forms revealed by direct microscopic examination and stained smears can enable prompt identification of arthrosporic yeasts such as *Saprochaete *spp. The early preliminary diagnosis of *Saprochaete *systemic infections may help clinicians ensure appropriate therapeutic management by using amphotericin B or voriconazole as the gold standard treatment for *S. capitata *IFI.

## References

[1] El Zein S, Hindy JR, Kanj SS (2020). Invasive saprochaete infections: an emerging threat to immunocompromised patients. Pathogens.

[2] De Hoog GS, Smith MT (2004). Ribosomal gene phylogeny and species delimitation in Geotrichum and its teleomorphs. Stud Mycol.

[3] Pfaller MA, Diekema DJ, Merz WG (2009). Infections caused by non-Candida, non-Cryptococcus yeasts. Clin Mycol.

[4] Alobaid K, Abdullah AA, Ahmad S, Joseph L, Khan Z (2019). Magnusiomyces capitatus fungemia: The value of direct microscopy in early diagnosis. Med Mycol Case Rep.

[5] Mazzocato S, Marchionni E, Fothergill AW (2015). Epidemiology and outcome of systemic infections due to saprochaete capitata: case report and review of the literature. Infection.

[6] Gurgui M, Sanchez F, March F (2011). Nosocomial outbreak of Blastoschizomyces capitatus associated with contaminated milk in a haematological unit. J Hosp Infect.

[7] D'Assumpcao C, Lee B, Heidari A (2018). A case of Magnusiomyces capitatus peritonitis without underlying malignancies. J Investig Med High Impact Case Rep.

[8] Schuermans C, van Bergen M, Coorevits L, Verhaegen J, Lagrou K, Surmont I, Jeurissen A (2011). Breakthrough Saprochaete capitata infections in patients receiving echinocandins: case report and review of the literature. Med Mycol.

[9] Cavanna C, Lallitto F, Mangione F, Tamarozzi F, Marone P, Ceriana P (2017). Fungemia due to Saprochaete capitata in a non-neutropenic patient hospitalized in an intensive care unit after cardiac surgery. J Mycol Med.

[10] Bariş A, Gencer H, Dalgiç N, Şik G, Özakkaş F, Aktaş E (2017). Magnusiomyces capitatus peritonitis in a child with acute lymphocytic leukemia as a breakthrough infection during caspofungin therapy. Pediatr Infect Dis J.

